# ROR2 increases the chemoresistance of melanoma by regulating p53 and Bcl2-family proteins via ERK hyperactivation

**DOI:** 10.1186/s11658-022-00327-7

**Published:** 2022-03-08

**Authors:** María Victoria Castro, Gastón Alexis Barbero, Paula Máscolo, Rocío Ramos, María Josefina Quezada, Pablo Lopez-Bergami

**Affiliations:** 1 Centro de Estudios Biomédicos, Básicos, Biotecnológicos, Aplicados y Desarrollo (CEBBAD), Universidad Maimónides, Hidalgo 775, 6th Floor, Lab 602, 1405 Buenos Aires, Argentina; 2 Consejo Nacional de Investigaciones Científicas y Técnicas (CONICET), 1425 Buenos Aires, Argentina

**Keywords:** ROR2, ERK, Melanoma, Chemoresistance, Apoptosis

## Abstract

**Background:**

ROR2 is a tyrosine-kinase receptor whose expression is dysregulated in many human diseases. In cancer, ROR2 stimulates proliferation, survival, migration, and metastasis, and is associated with more aggressive tumor stages. The purpose of this work is to study the role of ROR2 in the chemoresistance of melanoma.

**Methods:**

Gain- and loss-of-function experiments were used to study the biological function of ROR2 in melanoma. Cell death induced by chemotherapeutic drugs and BH-3 mimetics was evaluated using crystal violet cytotoxicity assays and annexin V/propidium iodide staining. Western blots were used to evaluate the expression of proteins implicated in cell death. The differences observed between cells with manipulation of ROR2 levels and control cells were evaluated using both Student’s *t*-test and ANOVA.

**Results:**

We describe that ROR2 contributes to tumor progression by enhancing the resistance of melanoma cells to both chemotherapeutic drugs and BH-3 mimetics. We demonstrate that ROR2 reduced cell death upon treatment with cisplatin, dacarbazine, lomustine, camptothecin, paclitaxel, ABT-737, TW-37, and venetoclax. This effect was mediated by the inhibition of apoptosis. In addition, we investigated the molecular mechanisms implicated in this role of ROR2. We identified the MDM2/p53 pathway as a novel target of ROR2 since ROR2 positively regulates MDM2 levels, thus leading to p53 downregulation. We also showed that ROR2 also upregulates Mcl-1 and Bcl2-xL while it negatively regulates Bax and Bid expression. The effect of ROR2 on the expression of these proteins is mediated by the hyperactivation of ERK.

**Conclusions:**

These results demonstrate that ROR2 contributes to melanoma progression by inhibiting apoptosis and increasing chemoresistance. These results not only position ROR2 as a marker of chemoresistance but also support its use as a novel therapeutic target in cancer.

**Supplementary Information:**

The online version contains supplementary material available at 10.1186/s11658-022-00327-7.

## Background

Melanoma is a malignant tumor of melanocytes and the most lethal of skin tumors. In the last 50 years, its incidence has risen faster than that of almost any other cancer, and in 2020 there were 100,350 newly diagnosed melanomas and 6850 deaths worldwide [1]. Melanoma can be removed via surgical resection in patients with early diagnosis; however, owing to its high metastatic potential, patients with advanced malignancies exhibit poor prognoses. At the molecular level, melanoma is characterized by the highly prevalent BRAF^V600E^ mutation that renders the MAPK/ERK pathway constitutively active and is critical for melanoma progression. Many other signaling pathways such as PI3K/Akt, PKC, STAT3, Wnt, and Eph/ephrin are also constitutively activated [2–6].

Cytotoxic chemotherapy (CC) has an established record of success against many different cancers but has proven ineffective against melanoma. Melanoma is intrinsically resistant to diverse cytotoxic insults, such as DNA damage (e.g., by irradiation, alkylation, methylation, or crosslinking), microtubule destabilization, or topoisomerase inhibition. A major mechanism of resistance is a profound dysregulation of cell death pathways due to the aberrant expression of inhibitors of apoptosis proteins (IAP), FLIP, p53, Fas, caspases, and Bcl2-family proteins, among others [7]. Despite its inefficacy, CC was the standard-of-care for melanoma patients until the advent of targeted therapy and immune checkpoint inhibitors (ICI) during the last decade. These two approaches have provided unprecedented advances in patient outcomes, improving the 5-year survival to almost 50% [8]. However, many patients are not benefited owing to innate and acquired resistance, thus establishing the need to identify novel targets to improve melanoma therapy.

ROR2 is a tyrosine kinase receptor of Wnt5a that plays a major role during embryonic development but is strongly downregulated after birth [9]. ROR2 aberrantly re-expresses in several pathological conditions in the adult. Interestingly, ROR2 possesses dual roles in cancer and can either suppress or promote carcinogenesis in different types of tumors [10, 11]. In the latter cases, ROR2 is usually upregulated and its elevated expression promotes increases tumorigenic properties, such as proliferation, migration, invasion, anchorage-independent growth, epithelial-to-mesenchymal transition (EMT), and in vivo tumor development [9, 11, 12]. It is noteworthy that, despite the established connection between EMT and drug resistance in cancer [13], the participation of ROR2 in apoptosis and chemoresistance has been poorly described [9–11]. The dual role of ROR2 was demonstrated in melanoma, where ROR2 inhibits proliferation and increases migration, invasion, and metastasis [14–16]. Following our recent observation that ROR2 potently induces EMT in melanoma [17], our goal was to evaluate the participation of ROR2 in the chemoresistance of melanoma.

## Methods

### Cell culture

Melanoma cells lines used in this work were provided by Dr. Zeev Ronai (Sanford Burnham Prebys Medical Discovery Institute), except for the M2 cell line, which was provided by Sergio Alvarez (IMIBIO-CONICET) [16]. Cells were maintained in DMEM supplemented with 10% fetal bovine serum (FBS; Gibco), 100 U/ml penicillin, and 100 mg/ml streptomycin (Invitrogen), at 37 °C and 5% CO_2_.

### shRNA constructs, overexpression system, and viral infection

Silencing of ROR2 was achieved by stable transduction of cells with plasmids encoding ROR2-specific short-hairpin RNA (shRNA) C2 (5′-CTGGGTGTATGCCCTCATGAT-3′) and C4 (5′-CCCTGGTGCTTTACGCAGAAT-3′) in the MeWo cell line and shRNA C4 in the M2 cell line. Both shRNA C2 and C4 similarly inhibited ROR2 expression and were validated in a previous publication from our laboratory [14]. Human ROR2 cDNA was cloned into the VIRSP vector. Generation of both retroviral (ROR2 shRNA) and lentiviral (ROR2 cDNA) particles and stable transduction were performed as described [5].

### Crystal violet cytotoxicity assay

The assay was performed as described [16]. Briefly, cells (5 × 10^3^ per well) were plated in 96-well plates and incubated for 24 h with DMEM 10% FBS. Thereafter, cisplatin (40 µM, Santa Cruz Biotechnology, Santa Cruz CA), paclitaxel (10 µM, Sigma-Aldrich, St. Louis, MI), lomustine (75 µM for A375 and UACC903 or 100 µM for M2 and MeWo, Chemovet, Buenos Aires, Argentina), dacarbazine (20 µM, Sigma-Aldrich), camptothecin (1 µM, Santa Cruz Biotechnology), ABT-737 (5 µM, Santa Cruz Biotechnology), TW-37 (5 µM, Santa Cruz Biotechnology), or venetoclax (10 µM, MedChem Express) were added to the plate (in quadruplicates) and left for 48 h. Cells were incubated with DMSO as a control. After removal of the medium, the plates were fixed and stained with crystal violet for 30 min. After rinsing, crystal violet was dissolved in 10% acetic acid and the absorbance [optical density (OD)] was detected at 590 nm with a µQuant microplate reader (Biotek Instruments). A plate fixed at 6 h was used as a control of seeding. A standard calibration curve was used to convert OD to the number of cells. The percentage of cytotoxicity was calculated as the quotient between the number of cells in treated wells and the number of cells in untreated wells times 100. Dose–response experiments were performed beforehand to select drugs concentrations that induced around 50% cytotoxicity.

### Quantification of apoptotic cell death

The cells were seeded on 60 mm plates at a density of 1.25 × 10^5^ cells per well. The following day, the cells were treated with lomustine as indicated above. Cells were washed twice with PBS, filtered, and resuspended in 100 µL of annexin V binding buffer (pH 7.4) (BD Biosciences, Franklin Lakes, NJ, USA). Then, annexin V-Alexa Fluor 488 (BD Biosciences) was added and incubated for 15 min under dark conditions. Propidium iodide (0.1 µg/mL; Sigma-Aldrich; Merck KGaA, Darmstadt, Germany) was added just prior to signal acquisition. Cells were analyzed using a FACSAria flow cytometer (BD Biosciences, San Jose, CA) and analyzed with FACSDiva 7.6.1 software (BD Biosciences) [18].

### Western blotting

For the Western blotting analysis, cell lysates were collected by the addition of lysis buffer supplemented with protease and phosphatase inhibitors as described [19]. Between 20 and 50 μg of proteins were diluted in 6× Lemmli buffer, boiled at 95 °C for 5 min, separated on 8–12% SDS–PAGE gels, and then transferred to nitrocellulose membrane. The membranes were blocked with 5% milk in 0.05% Tween-PBS at room temperature for 1 h and then incubated with the primary antibodies at 4 °C overnight. The following antibodies were used: GAPDH (sc-25,778), p53 (sc-126), MDM2 (sc-965), Bcl-xL (sc-634), Bid (sc-6538), and Bax (sc-7480) from Santa Cruz Biotechnologies; Mcl-1 (cs-5453), p-ERK (cs-9106), and ERK (cs-9102) from Cell Signaling (Danvers, MA); and β-actin (A5441) from Sigma. The primary antibody anti-ROR2 [34] was from QED Bioscience. The corresponding HRP-conjugated secondary antibodies, anti-mouse (Bio-Rad, 170-6515), anti-rabbit (Bio-Rad, 170-6516), or anti-goat (sc-2020), were incubated for 1 h at room temperature. Immunoreactive bands were detected by an ECL system (Amersham Biosciences, UK) using an image reader (ImageQuant 350, GE Healthcare, Chicago, IL, USA). Quantification of band intensities was performed using ImageJ (NIH). The intensity of each band was normalized to GAPDH or β-actin, and the fold change (FC) relative to control cells was calculated. To draw a conclusion on a particular experiment, at least three biological (independent) replicates of paired samples were examined to calculate the mean and standard deviation. The log transformation of FC values was calculated to obtain a more symmetric distribution that better suits the normality assumptions of the subsequent statistical tests.

### Immunofluorescence

Cells were seeded in slides and 48 h later were fixed in DMEM with 4% PFA for 10 min at room temperature (RT). Slides were washed in PBS and placed in permeabilization solution (0.5% Triton-X 100, 3 mM Cl_2_Mg, 6.84% sucrose) on ice for 5 min. Then, slides were blocked in PBS 3% BSA for 1 h at RT. Next, the slides were incubated with MDM2 (sc-965) antibody, overnight at 4 °C in a humidified chamber. After the incubation, the slides were washed three times with PBS, and incubated with secondary antibody (Alexa Fluor 488) for 1 h in the dark at RT. Finally, slides were mounted with Vectashield DAPI ANTIFADE Mounting Medium (Vector). Pictures were taken on a Nikon C1 Plus microscope and analyzed using FIJI.

### Statistics

All experiments were performed at least three times. Student’s *t*-test and ANOVA were performed to compare groups. Values of *p* < 0.05 were considered statistically significant. Statistical analyses were conducted using software from Graph-Pad Prism. The number of independent experiments and specific statistical analyses used in each experiment are indicated in the figure legends.

## Results

**Fig. 1:**
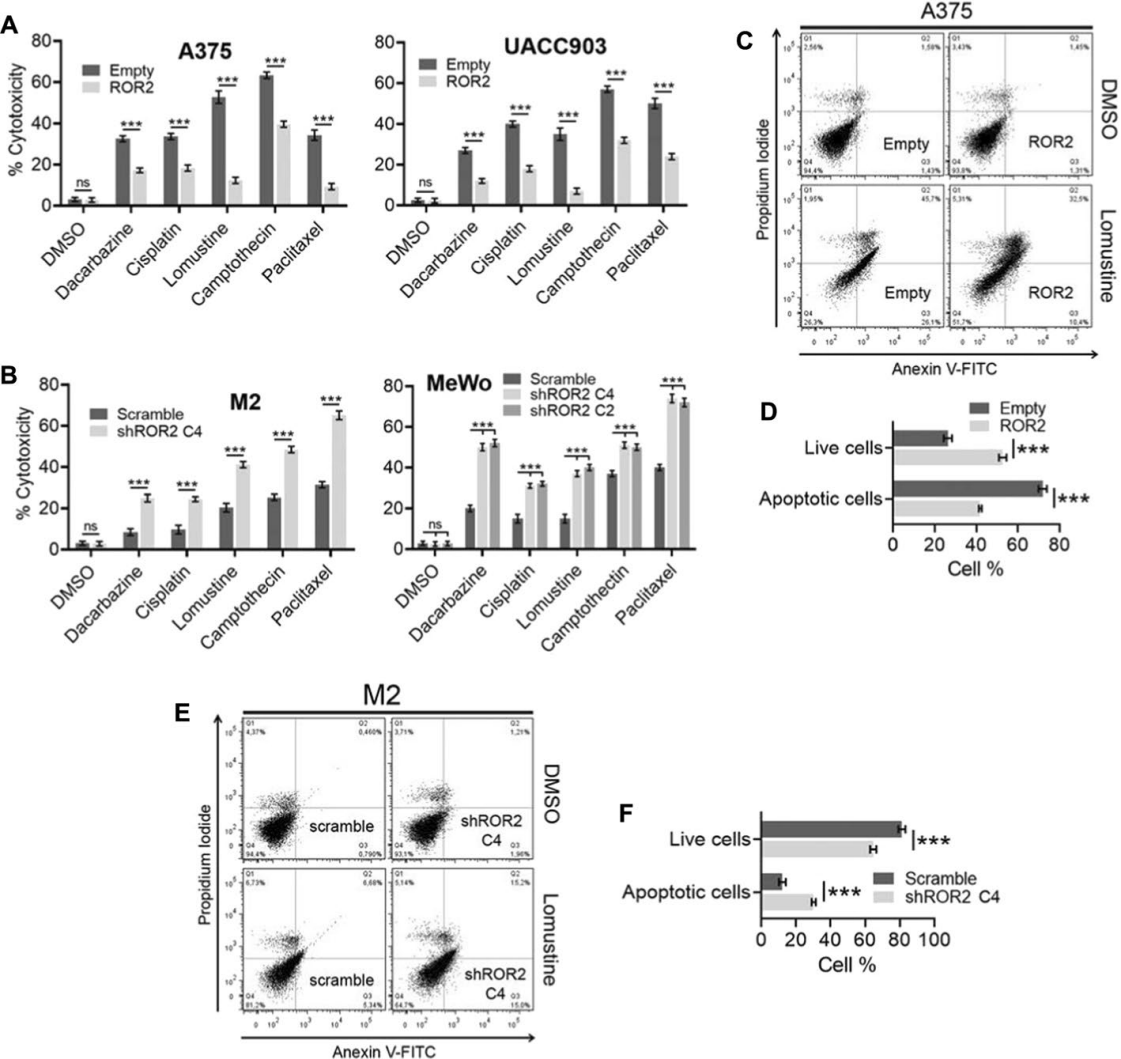
ROR2 enhances resistance of melanoma cells to chemotherapy drugs. **A** ROR2 overexpression increased cell survival in melanoma cells upon treatment with chemotherapeutic drugs. A375-empty, A375-ROR2, UACC903 empty, and UACC903-ROR2 were treated with cisplatin, paclitaxel, lomustine, dacarbazine, and camptothecin as indicated in Methods, and a crystal violet cytotoxicity assay was performed. Bar graph shows the mean ± standard deviation (SD) (*n* = 3) of the percent of cytotoxicity. The percentage of cytotoxicity was calculated as the quotient between the number of cells in treated wells and the number of cells in nontreated wells times 100. **B** ROR2 silencing enhances cell death induced by chemotherapeutic drugs. The experiment was performed as in **A** using M2-scramble, M2-shROR2, MeWo-scramble, and MeWo-shROR2 cells. **C** ROR2 prevents apoptosis by lomustine treatment. A375 empty and A375-ROR2 cells were treated with 75 µM lomustine for 48 h, stained with PI/annexin V, and analyzed by flow cytometry. Representative dot plots are shown. **D** Bar graphs show the mean ± SD (*n* = 3) of the percentage of live (Q4) and apoptotic (Q2 + Q3) cells from the experiment in **C**. **E**, **F** ROR2 silencing increased apoptosis by lomustine treatment. The experiment was performed as in **C** and **D** using M2-scramble and M2-shROR2 cells treated with 100 µM lomustine for 48 h. Statistical significance was tested by a one-tailed Student’s *t*-test or ANOVA (MeWo) (*n* = 3). *p < 0.01, **p < 0.001, ***p < 0.0001, *n* = 3. *n.s.* not significant

To address the effect of ROR2 expression in chemoresistance, we evaluated the cytotoxicity induced by five anticancer agents in melanoma cells with forced expression of ROR2. The cell lines A375 and UACC903 that express low levels of endogenous ROR2 [16] were selected for these experiments (Additional file 1: Fig. S1A). We assayed three DNA-damaging agents (cisplatin, dacarbazine, and lomustine), a topoisomerase inhibitor (camptothecin), and a microtubule inhibitor (paclitaxel). Following treatment with each of these drugs, both A375 and UACC903 cells overexpressing ROR2 presented reduced cell death compared with control cells (A375-empty and UACC903-empty) (Fig. 1A). Next, we assayed the effect of these drugs on loss-of-function experiments. To this end, we used the cell lines M2 and MeWo where the robust endogenous ROR2 expression was inhibited by RNA interference (Additional file 1: Fig. S1B). ROR2 knockdown using the ROR2 shRNA C4 sensitized M2 cells to the five tested compounds (Fig. 1B). Similar results were observed in MeWo cells upon silencing of ROR2 with two different shRNA (C4 and C2, Fig. 1B). To confirm these findings, we performed an annexin V-PI assay following treatment of the cells with lomustine, a nitrosourea usually prescribed to treat melanoma brain metastasis. Overexpression of ROR2 protected A375 cells from apoptosis induced by lomustine (Fig. 1C, D). In contrast, ROR2 silencing induced an increase in the number of apoptotic cells upon the cytotoxic stimuli (Fig. 1E, F). These results indicate that ROR2 promotes chemoresistance of melanoma cells to cytotoxic compounds with different mechanisms of action by inhibiting apoptosis.

**Fig. 2:**
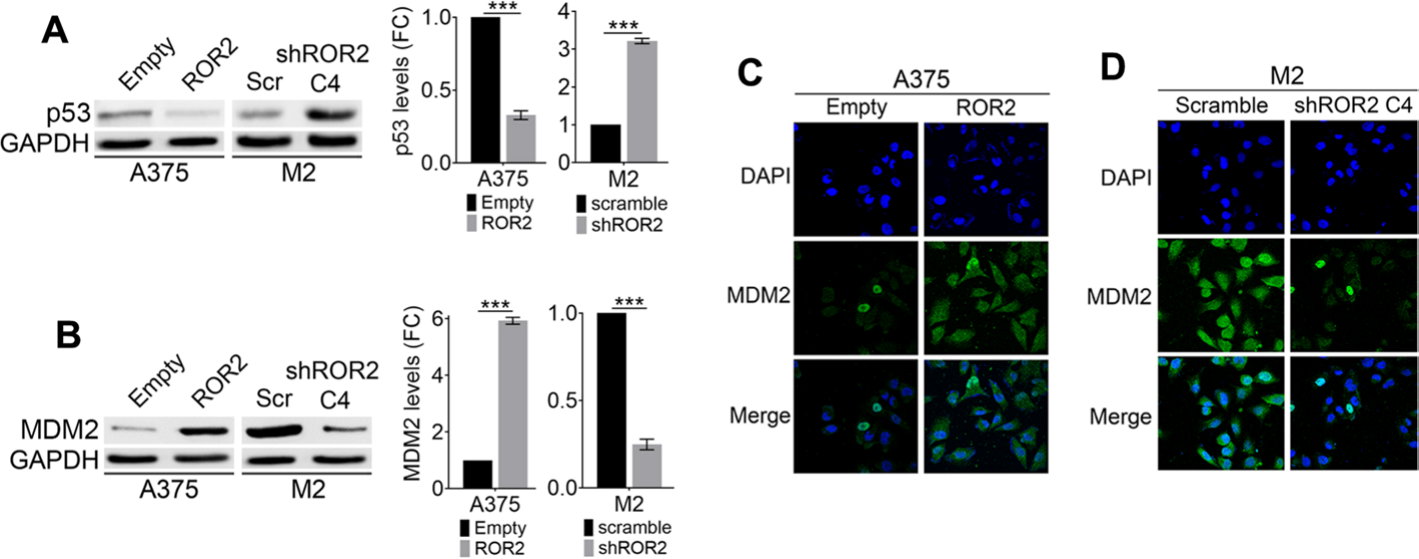
ROR2 increased MDM2 and decreased p53 protein levels. ROR2 regulates p53 **A** and MDM2 **B** levels. Protein extracts from the indicated cell lines were probed with the indicated antibodies. GAPDH was used as loading control. The blots displayed are representative of three independent experiments. Bar graphs show the mean ± SD (from three independent experiments) of p53 **A** or MDM2 **B** levels normalized to the loading control and expressed as the fold change relative to control cells. Statistical significance was tested by a one-tailed Student’s *t*-test, ***p < 0.0001, *n* = 3. **C**, **D** Assessment of MDM2 localization by immunofluorescence upon ROR2 overexpression (**C**) or silencing (**D**). A375-Empty and A375-ROR2 (**C**) or M2-scramble and M2-shROR2 (**D**) were stained with DAPI and anti-MDM2 antibody

We next investigated the molecular mechanisms mediating ROR2 effects on chemoresistance. Since reduced or abolished p53 function has been linked to resistance toward standard chemotherapy regimes in different cancers [20], including melanoma [21], we investigated p53 levels in cells with ROR2 gain- and loss-of-function. ROR2 overexpression decreased p53 levels, whereas ROR2 silencing increased p53 expression (Fig. 2A). We next measured changes in MDM2, a major p53 regulator. Forced expression of ROR2 in A375 cells increased MDM2 levels (Fig. 2B) and favored its cytoplasmic localization (Fig. 2C). In contrast, ROR2 silencing in M2 cells reduced MDM2 expression (Fig. 2B) that presented a restricted nuclear localization (Fig. 2D). These results are in agreement with the observed effect of ROR2 in cell death and indicate that one of the mechanisms by which ROR2 promotes chemoresistance is the upregulation of MDM2 and the downregulation of p53.

**Fig. 3:**
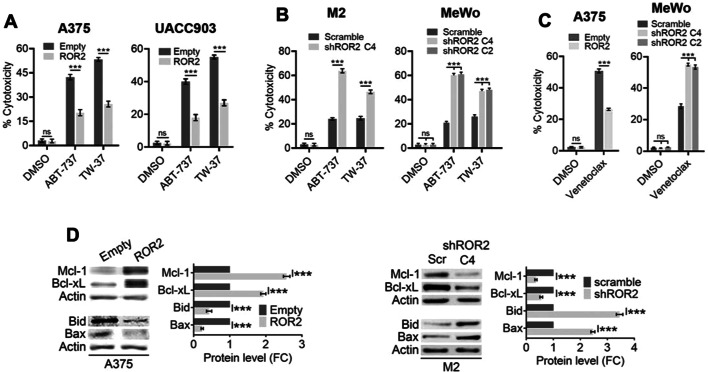
ROR2 enhances resistance of melanoma cells to BH-3 mimetics. **A** ROR2 overexpression increased cell survival in melanoma cells upon treatment with BH-3 mimetics. A375-empty, A375-ROR2, UACC903-empty, and UACC903-ROR2 were treated with 5 µM ABT-737 and 5 µM TW-37 for 48 h, and a crystal violet cytotoxicity assay was performed. Bar graph shows the mean ± SD (*n* = 3) of the percent of cytotoxicity. The percentage of cytotoxicity was calculated as the quotient between the number of cells in treated wells and the number of cells in nontreated wells times 100. **B** ROR2 silencing enhances cell death induced by BH-3 mimetics. The experiment was performed as in **A** using M2-scramble, M2-shROR2, MeWo-scramble, and MeWo-shROR2 cells treated with 10 µM ABT-737 and 5 µM TW-37. **C** ROR2 silencing enhances cell death induced by a Bcl2-specific BH-3 mimetic. The experiment was performed as in **A** using A375-empty, A375-ROR2, MeWo-scramble, and MeWo-shROR2 cells treated with 10 µM venetoclax. **D**, **E** ROR2 regulates expression of Bcl2-family proteins. Protein extracts from A375-empty and A375-ROR2 (**D**) and M2-scramble and M2-shROR2 (**E**) were probed with the indicated antibodies. β-Actin were used as loading control. The blots displayed are representative of three independent experiments. The graphs show the mean ± SD of each protein’s levels normalized to the corresponding loading control and expressed as the fold change (FC) relative to the corresponding control cell line (empty or scramble). Statistical significance was tested by a one-tailed Student’s *t*-test or ANOVA (MeWo), ***p < 0.0001, *n* = 3

A feature common to all five cytotoxic compounds used above is that they rely on functional p53 to induce apoptosis. We next wanted to determine whether ROR2 can also increase the resistance to cytotoxic drugs with other mechanisms of action. To address this point, we used BH-3 mimetics since they induce apoptosis independently of p53 [22]. BH3 mimetics are small molecules that mimic BH3 proteins by binding to anti-apoptotic Bcl2-family proteins and inhibiting their function. Therefore, we evaluated the effect of the BH-3 mimetics ABT-737 and TW-37 upon either overexpression or silencing of ROR2. ABT-737 binds with high affinity to Bcl2, Bcl‐xL, and Bcl-w but does not inhibit Mcl-1, Bfl-1, or Bcl2L10 [23]. TW-37 is a BH3-mimetic that targets primarily Mcl-1 [24]. Expression of ROR2 in both A375 and UACC903 cells reduced cell death upon treatment with both ABT-737 and TW-37 (Fig. 3A). Using the opposite approach, we observed that ROR2 silencing sensitized both M2 and MeWo cells to BH-3 mimetics (Fig. 3B). These results were confirmed by using venetoclax (ABT-199), a BH-3 mimetic selective for Bcl2. ROR2 overexpression in A375 cells inhibited venetoclax-induced cell death, whereas ROR2 silencing in MeWo cells enhanced cytotoxicity by this drug (Fig. 3C). To identify the mechanism by which ROR2 enhanced the resistance to BH-3 mimetics, we determined changes in the levels of Bcl2-family proteins. Cells overexpressing ROR2 present increased levels of the anti-apoptotic Bcl2 proteins Mcl-1 and Bcl-xL and decreased levels of Bcl2 pro-apoptotic proteins Bax y Bid (Fig. 3D). Silencing of ROR2 in M2 cells decreased the expression of Mcl1 and Bcl-xL and increased the expression of Bax y Bid (Fig. 3E). These results indicate that ROR2 regulates the expression of Bcl2-family proteins, thus contributing to chemoresistance.

**Fig. 4:**
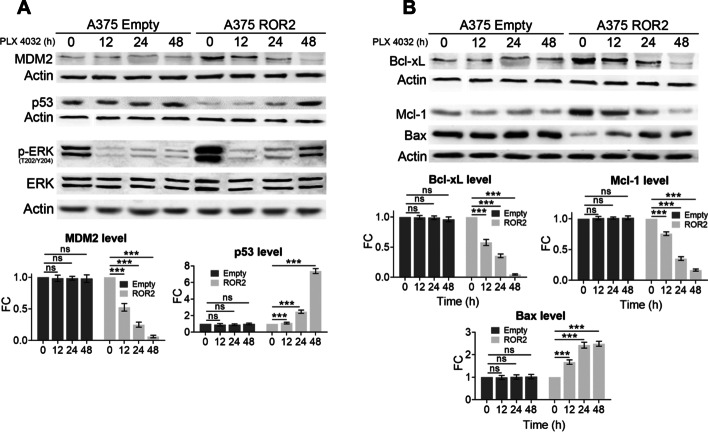
ROR2 regulates the expression of MDM2, p53, and Bcl2-family proteins through the hyperactivation of ERK. **A** PLX inhibited MDM2 levels and increased p53 in A375-ROR2 cells. The cells were treated with 10 µM PLX for the indicated time. The graphs show the mean ± SD of each protein’s levels normalized to the corresponding loading control and expressed as the fold change (FC) relative to untreated cells. **B** Bcl2 proteins are regulated by the MAPK/ERK pathway in A375-ROR2 cells. The cells were treated with 10 µM PLX for the indicated times. The graphs show the mean ± SD of Bcl-xL, Mcl-1, and Bax normalized to the corresponding loading control and expressed as the fold change (FC) relative to untreated cells. Statistical significance was tested by a one-tailed Student’s *t*-test or ANOVA as appropriate (*n* = 3). *p < 0.01, **p < 0.001,***p < 0.0001, *n.s.* not significant

We have recently demonstrated that a major consequence of ROR2 expression in melanoma is the hyperactivation of the MAPK/ERK pathway [17]. Considering this pathway is a major player in the chemoresistance of cancer cells [25], we wanted to determine whether the regulation of pivotal regulators (i.e., p53 and Bcl2 proteins) of chemoresistance by ROR2 is mediated by activation of the MAPK/ERK pathway. To this end, we used the BRAF^V600E^ inhibitor (BRAFi) PLX-4032 (PLX), that inhibits both basal ERK activity and hyperactive ERK signal in A375-empty and A375-ROR2 cells, respectively (Fig. 4A) [15]. Incubation with PLX for up to 48 h did not alter the expression levels of either MDM2 or p53 in A375 control cells (A375-empty, Fig. 4A). In contrast, in A375-ROR2 cells, MDM2 levels decreased in a time-dependent manner following treatment with PLX. In agreement with this observation, p53 levels increased almost eightfold upon 48 h of PLX treatment in A375-ROR2 cells (Fig. 4A). Similar to what was described for MDM2, PLX inhibited the expression of both Bcl-xL and Mcl-1 in A375-ROR2 cells but not in A375-empty cells (Fig. 4B). The downregulation of the pro-apoptotic protein Bax by ROR2 was also mediated by the activation of the MAPK/ERK pathway since PLX markedly increased Bax levels in A375-ROR2 cells (Fig. 4B). Altogether, our data demonstrate that ROR2 inhibits apoptosis and promotes chemoresistance of melanoma cells by hyperactivating ERK, which in turn upregulates MDM2, Mcl-1, and Bcl-xL, and downregulates p53, Bax, and Bid.

## Discussion

ROR2 is a tyrosine-kinase receptor that has an intriguing dual role in cancer by either suppressing or promoting carcinogenesis in different tumor types. The processes more often regulated by ROR2 in cancer are proliferation, invasion, and migration. Still, in some tumor types, ROR2 expression has been associated with altering the cell’s response to cytotoxic drugs. Two articles have linked ROR2 expression with resistance to platinum in ovarian cancer. It was shown that both ROR1 and ROR2 expression is increased in ovarian cancer cells resistant to cisplatin and that simultaneous knockdown of both receptors sensitized cells to this drug [26]. Moreover, ROR2 expression is increased in ovarian cancer patients with platinum resistance [27]. Similarly, ROR2 expression is increased in colon cancer cells resistant to histone deacetylase inhibitors [28]. Studies in prostate cancer cells revealed a complex role of ROR2 in the resistance to taxanes. ROR2 expression is increased in cabazitaxel-resistant cells but not in docetaxel-resistant cells. Further, ROR2 silencing reverted cabazitaxel resistance. However, two genetically related (RWPE-1 and RWPE-2) prostate cancer cell lines that express low versus high levels of ROR2 were similarly sensitive to cabazitaxel [29]. In stark contrast with these observations, ROR2 overexpression increased the chemosensitivity to doxorubicin in the esophageal squamous cell carcinoma cell line KYSE150 [30]. Importantly, none of the works cited above has investigated the biological processes or the molecular mechanisms by which ROR2 regulates the response to cytotoxic drugs.

In the present work, we established that ROR2 promotes chemoresistance to several cytotoxic drugs and determined that ROR2 exerts this effect by inhibiting apoptosis. The role of ROR2 in the apoptotic response following cytotoxic insults has not been studied to date. Instead, ROR2 was shown to induce cell proliferation and suppress basal apoptosis in breast cancer [31] and renal cell carcinoma [32]. As is often observed with ROR2, the opposite result has been described in other tumor types, since overexpression of ROR2 repressed the proliferation of high-grade serous ovarian cancer cells and induced cell apoptosis in ovarian cancer [33] and gastric cancer [34]. However, since these experiments were conducted in the absence of a death stimulus, the observed differences in apoptosis in these studies are, predictably, of small magnitude and not necessarily indicative of a role of ROR2 in the response against cytotoxic drugs.

Malignant melanoma is highly refractory to anticancer drugs despite retaining wild-type p53 [35]. Thus, any mechanism that inhibits p53 could impair the connection between DNA damage (caused by chemotherapeutic agents) and the triggering of apoptosis. We have discovered that ROR2 exerts its effect by regulating the levels of p53 and of Bcl2-family proteins. We demonstrated that ROR2 decreased p53 by promoting an increase in MDM2 levels, a major negative regulator of p53. To our knowledge, the regulation of p53 by ROR2 has not been described to date. The regulation of Bcl2 proteins by ROR2 can be also mediated by MDM2 since it was shown that MDM2 can regulate Bax and Bcl2 in a negative and positive fashion, respectively [36]. ROR2 was previously shown to upregulate survivin 1, Bcl2, and Bcl-xL in breast cancer cells, although the underlying mechanisms were not investigated [31].

Here, we described that the regulation of both p53 and Bcl2-family members by ROR2 is mediated by the hyperactivation of ERK. We have previously demonstrated that ERK hyperactivation is critical for the induction of EMT by ROR2 [17]. EMT is a cell-biological process in which epithelial cells with apical–basal polarity undergo cytoskeletal rearrangement to become motile mesenchymal cells. This process is thought to contribute to chemoresistance and metastasis. Our observation suggests that both processes, EMT and chemoresistance, are regulated by a ROR2/ERK pathway. Thus, targeting ROR2 could inhibit both processes that are profoundly connected with melanoma outcomes.

Despite the improved efficacy of both targeted therapy and immunotherapy, CC remains a treatment option in metastatic melanoma, where it has moved from a first-line to a second- or higher-line treatment strategy. The search for better therapeutic strategies has renewed the interest in chemotherapy since it has become evident that they can be useful in the new biological scenarios generated upon BRAFi and ICI administration. In the past years, several investigators have shown that melanoma patients that received CC after progressing on immunotherapies presented promising responses [37–42]. Inversely, in vitro and in vivo data suggest that the immunomodulatory effects of CC may potentiate the effects of immunotherapy [43, 44]. Since a common BRAF resistance mechanism involves the reactivation of MEK/ERK signaling [45], the activation of ERK by ROR2 suggests that ROR2 might also enhance resistance to BRAFi. Thus, activation of the ROR2/ERK pathways mechanistically explains the prior observation that ROR2 silencing sensitized melanoma cells to vemurafenib [46]. Along this line, CC appears to combine efficiently with anti-BRAF therapies [47, 48].

These findings show that ROR2 can be used as a predictor of chemoresistance against cytotoxic chemotherapy but also suggest that increased ROR2 levels can contribute to both intrinsic and acquired resistance of melanoma to immune and targeted therapeutics. Along this line, ROR2 has been identified as a part of a transcriptional signature found in PD-1 innately resistant tumors [49]. Thus, despite the dual role of ROR2 in melanoma, our results indicate that this tyrosine kinase receptor can be a therapeutic target for advanced melanoma. Along this line, antibody-based therapeutic tools targeting ROR2 have been developed and are being tested in clinical trials against cancer [11].

## Conclusions

Overall, our results demonstrate that ROR2 is a major regulator of the expression levels of both p53 and Bcl2-family proteins through the hyperactivation of ERK. Using this mechanism, ROR2 contributes to melanoma progression by inhibiting apoptosis and increasing chemoresistance. Thus, the elevated ROR2 expression can serve as both a marker of chemoresistance and a therapeutic target melanoma.

## Supplementary Information

**Additional file 1: Fig. S1.** Overexpression and silencing of ROR2 in melanoma cells. **A** Overexpression of ROR2 in A375 and UACC903 cell lines. ROR2 expression was assessed by western blot in cells stably transduced with either control (empty) or a ROR2-expressing plasmid. GAPDH was used as a loading control. **B** Silencing of ROR2 in M2 and MeWo cells. ROR2 expression was assessed by western blot in cells stably transduced with either control (scramble) or two shRNA for ROR2 (C4 and C2). GAPDH was used as a loading control.

## Data Availability

All data generated or analyzed during this study are included in this published article.

## Abbreviations

CC: Cytotoxic chemotherapy
ICI: Immune checkpoint inhibitors
EMT: Epithelial-to-mesenchymal transition
BRAFi: BRAF^V600E^ inhibitor
PLX: PLX-4032
