# Correction to: ROR2 increases the chemoresistance of melanoma by regulating p53 and Bcl2-family proteins via ERK hyperactivation

**DOI:** 10.1186/s11658-022-00357-1

**Published:** 2022-07-03

**Authors:** María Victoria Castro, Gastón Alexis Barbero, Paula Máscolo, Rocío Ramos, María Josefna Quezada, Pablo Lopez-Bergami

**Affiliations:** 1 Centro de Estudios Biomédicos, Básicos, Biotecnológicos, Aplicados y Desarrollo (CEBBAD), Universidad Maimónides, Hidalgo 775, 6th Floor, Lab 602, 1405 Buenos Aires, Argentina; 2 Consejo Nacional de Investigaciones Científcas y Técni-Cas (CONICET), 1425 Buenos Aires, Argentina

## Correction to: Cellular & Molecular Biology Letters (2022) 27:23 https://doi.org/10.1186/s11658-022-00327-7

Following publication of the original article [1], the authors identified a few errors in panel A of Fig. 4. Two western blot images from panel B (Bcl-xL and Actin) were duplicated by mistake into panel A in place of the western blots for MDM2 and the Actin controls for both MDM2 and p53. The correct Fig. 4 is given in this correction article.

**Fig. 4:**
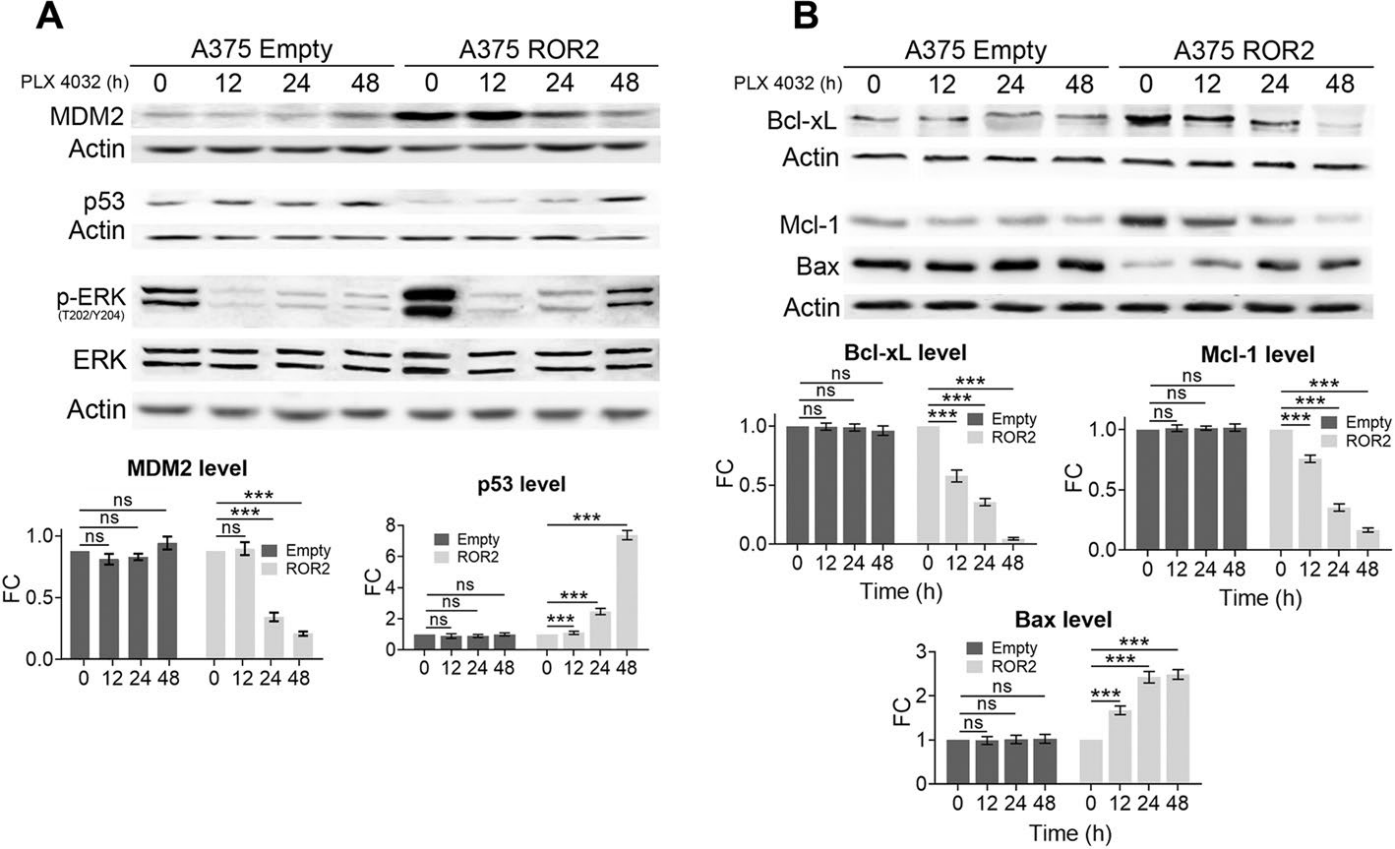
ROR2 regulates the expression of MDM2, p53, and Bcl2-family proteins through the hyperactivation of ERK. **A** PLX inhibited MDM2 levels and increased p53 in A375-ROR2 cells. The cells were treated with 10 µM PLX for the indicated time. The graphs show the mean ± SD of each protein’s levels normalized to the corresponding loading control and expressed as the fold change (FC) relative to untreated cells. **B** Bcl2 proteins are regulated by the MAPK/ERK pathway in A375-ROR2 cells. The cells were treated with 10 µM PLX for the indicated times. The graphs show the mean ± SD of Bcl-xL, Mcl-1, and Bax normalized to the corresponding loading control and expressed as the fold change (FC) relative to untreated cells. Statistical signifcance was tested by a one-tailed Student’s t-test or ANOVA as appropriate (n = 3). ***p < 0.0001, *n.s.* not significant

## References

[1] Castro MV, Barbero GA, Máscolo P, Ramos R, Quezada MJ, Lopez-Bergami P (2022). ROR2 increases the chemoresistance of melanoma by regulating p53 and Bcl2-family proteins via ERK hyperactivation. Cell Mol Biol Lett.

