# Supplementary figures and images for: ROR2 increases the chemoresistance of melanoma by regulating p53 and Bcl2-family proteins via ERK hyperactivation
Source: Cell Mol Biol Lett. 2022 Mar 8;27:23. doi: 10.1186/s11658-022-00327-7 (PMC8903712; doi:10.1186/s11658-022-00327-7)

Additional file 1: Fig. S1

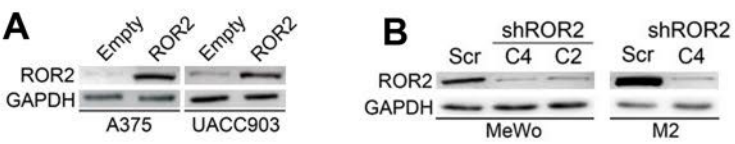

Supplement: Supplementary file 1 — Additional file 1: Fig. S1. Overexpression and silencing of ROR2 in melanoma cells. A Overexpression of ROR2 in A375 and UACC903 cell lines. ROR2 expression was assessed by western blot in cells stably transduced with either control (empty) or a ROR2-expressing plasmid. GAPDH was used as a loading control. B Silencing of ROR2 in M2 and MeWo cells. ROR2 expression was assessed by western blot in cells stably transduced with either control (scramble) or two shRNA for ROR2 (C4 and C2). GAPDH was used as a loading control. [file 11658_2022_327_MOESM1_ESM.pdf]
